# Draft Genome Sequence of *Mycobacterium peregrinum* Strain CSUR P2098

**DOI:** 10.1128/genomeA.01274-15

**Published:** 2015-11-05

**Authors:** Shady Asmar, Nicolás Rascovan, Catherine Robert, Michel Drancourt

**Affiliations:** Aix Marseille Université, URMITE, UMR 63, CNRS 7278, IRD 198, Inserm 1095, Faculté de Médecine, Marseille, France

## Abstract

*Mycobacterium peregrinum* is a nonpigmented rapid growing nontuberculosis species belonging to the *Mycobacterium fortuitum* group. The draft genome of *M. peregrinum* type I CSUR P2098 comprises 7,109,836 bp exhibiting a 66.23% G+C content, 6,894 protein-coding genes, and 100 predicted RNA genes. Its genome analysis suggests this species differs from *Mycobacterium senegalense*.

## GENOME ANNOUNCEMENT

Initially reported in 1962, *Mycobacterium peregrinum* (1) was officially described 30 years later as a rapidly growing nontuberculous mycobacterium in the *Mycobacterium fortuitum* complex (2, 3). It was further divided into pipemidic acid-susceptible *M. peregrinum* type I and pipemidic acid-resistant *M. peregrinum* type II (4), regarded as a human strain of *Mycobacterium senegalense* (5). *M. peregrinum* has been isolated from various water-related sources (6–8). *M. peregrinum* accounts for only 1% to 2% of infections due to rapid growing mycobacteria (9–13).

We performed whole-genome sequencing of *M. peregrinum* CSUR P2098 strain, a type I, in order to firmly ensure its taxonomic relationships within the *M. fortuitum* complex.

Genomic DNA was isolated from *M. peregrinum* CSUR P2098 cultured in MGIT Middlebrook liquid culture (Becton, Dickinson, Le Pont-de-Claix, France) at 37°C in a 5% CO_2_ atmosphere. *M. peregrinum* genomic DNA was sequenced by Illumina MiSeq runs (Illumina Inc., San Diego) using a 5-kb mate-paired library. Reads were trimmed using Trimmomatic (14), and assembled using Spades v3.5 (15, 16). Contigs were combined together by SSPACE v2 (17) and Opera v2 (18) helped by GapFiller v1.10 (19). This resulted in a draft genome consisting in six scaffolds and seven contigs for a total of 7,109,836 bp and a G+C content of 66.23%. Noncoding genes and miscellaneous features were predicted using RNAmmer (20), ARAGORN (21), Rfam (22), PFAM (23), and Infernal (24). Coding DNA sequences (CDSs) were predicted using Prodigal (25) and functional annotation was achieved using BLASTp against the GenBank database (26) and the Clusters of Orthologous Groups (COGs) database (27, 28). The genome was shown to encode at least 100 predicted RNA including six rRNA, 64 tRNA, one tmRNA, and 29 miscellaneous RNA. A total of 6,894 identified genes yielded a coding capacity of 7,054,578 bp (coding percentage, 99.22%). Among these genes, 5,101 (74%) were found as putative proteins and 1,282 (18.6%) were assigned as hypothetical proteins. Moreover, 4,020 (58.3%) genes matched a least one sequence in the COGs database with BLASTp default parameters. Further, *M. peregrinum* CSUR P2098 genome was incorporated into *in silico* DNA-DNA hybridation (DDH) (29) with the *M. fortuitum* complex species and other reference genomes selected on the basis of their 16S rRNA gene sequence proximity with *M. peregrinum* and DDH values were estimated using the GGDC v2.0 online tool (30). This analysis yielded 33.8% ± 2.47 similarity with *Mycobacterium septicum* DSM 44393 (31), 33.6% ± 2.47 *Mycobacterium fortuitum* ATCC 6841 (32), 33.5% ± 2.47 with *Mycobacterium* sp. VKM Ac-1817D (33), 32.3% ± 2.46 with *Mycobacterium senegalense* CK2 and *Mycobacterium neworleansense* ATCC 49404 (34), 31.8% ± 2.46 with *Mycobacterium conceptionense* D16 (unpublished data) (35), 31.6% ± 2.46 with *Mycobacterium farcinogenes* DSM43637 (36), and 20.7% ± 2.32 to *Mycobacterium gilvum* Spyr1 (37).

These data confirm *M. peregrinum* as a unique species within the *M. fortuitum* complex. In particular, these data suggest that *M. peregrinum* differs from *M. senegalense*.

### Nucleotide sequence accession numbers.

The *M. peregrinum* genome sequence has been deposited at EMBL under the accession numbers CYSH01000001 to CYSH01000007.
